# Recent Advances in Silk Fibroin-Based Composites for Bone Repair Applications: A Review

**DOI:** 10.3390/polym17060772

**Published:** 2025-03-14

**Authors:** Siyu Zhu, Qian Zhang, Xiang Xu, Zulan Liu, Guotao Cheng, Dingpei Long, Lan Cheng, Fangyin Dai

**Affiliations:** 1 State Key Laboratory of Resource Insects, Key Laboratory of Sericultural Biology and Genetic Breeding, Ministry of Agriculture and Rural Affairs, Southwest University, Chongqing 400715, China; zsy3492133451@163.com (S.Z.); zqqqqqqqqq@email.swu.edu.cn (Q.Z.); 13606798494@163.com (X.X.); lzlxndx2020@swu.edu.cn (Z.L.); cheng_2001@swu.edu.cn (G.C.); dplong@swu.edu.cn (D.L.); 2 College of Sericulture, Textile and Biomass Sciences, Southwest University, Chongqing 400715, China; 3 Yibin Academy of Southwest University, Yibin 644000, China

**Keywords:** silk fibroin, bone tissue, silk fibroin composite scaffolds, bone repair

## Abstract

Silk fibroin (SF), a natural high-molecular-weight fiber protein extracted from silk, has demonstrated immense potential in bone tissue repair and regeneration due to its exceptional physicochemical properties. Silk fibroin can be processed into various scaffold forms using diverse fabrication techniques, combined with other biomaterials to create composite structures, or chemically modified to address a wide range of bone defect conditions. This review provides a comprehensive examination of the role of silk fibroin and its composites in bone tissue engineering, with particular emphasis on preclinical studies investigating various silk fibroin-based composite scaffolds in osteogenesis. Additionally, it discusses the current status and challenges in preparing silk fibroin scaffolds tailored to bone tissue defects and explores innovative approaches such as silk fibroin membranes, hydrogels, and 3D-printed constructs. The review begins with an introduction to bone biology, including its composition, structure, healing mechanisms, and the development of bone repair materials. It then delves into the unique properties of silk fibroin, including its composition, structure, and physicochemical attributes, which make it an ideal candidate for bone tissue engineering. This review provides valuable insights into their design, fabrication, and application by critically analyzing recent advancements in silk fibroin-based scaffolds and their functional modifications. Finally, it offers a forward-looking perspective on the future development and translational potential of silk fibroin and its composites in the field of bone repair materials.

## 1. Introduction

**Figure 1:**
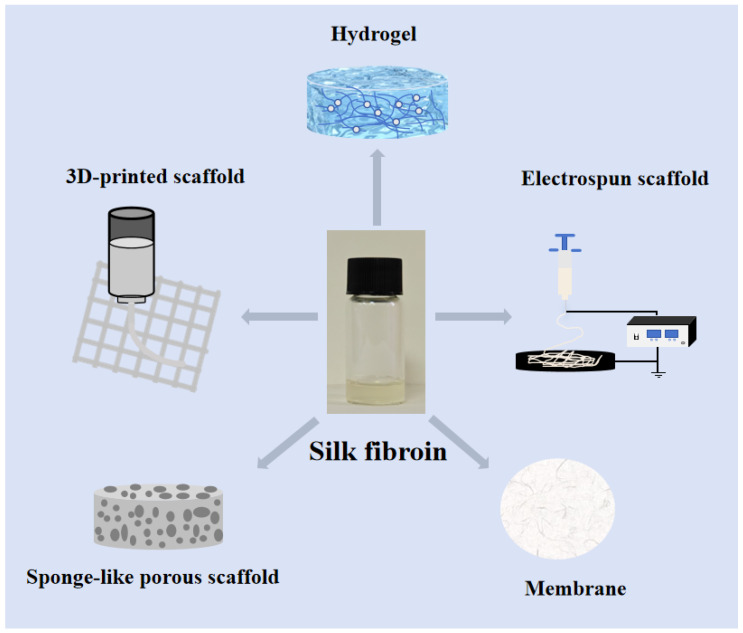
Types of silk fibroin scaffolds for bone repair.

Bone plays a crucial role in maintaining the structural framework of the human body, supporting mobility, protecting vital organs, and housing bone marrow for hematopoiesis. However, the increasing incidence of bone injuries caused by accidents, trauma, and degenerative diseases poses a significant challenge to global healthcare systems. Additionally, the aging population, driven by the growing proportion of older adults worldwide, has further amplified the prevalence of orthopedic disorders such as osteoporosis, osteoarthritis, and bone fractures. These trends have resulted in a substantial rise in the demand for effective bone repair and regeneration strategies [1]. For instance, in China, the market size for bone repair materials grew to approximately 2.02 billion yuan in 2018 and is projected to reach 5.34 billion yuan by 2023. Despite advances in clinical practices, autologous bone grafting remains the gold standard for bone regenerative repair due to its superior osteogenic potential and the absence of immune rejection [2]. However, this approach is far from ideal, as it is limited by donor site availability, significant morbidity, and graft failure rates of 50% in certain cases [3,4]. These challenges have underscored the need for alternative bone repair strategies that can overcome the limitations of autografts while providing efficient, scalable, and patient-specific solutions. In recent years, with the continuous development of bone tissue engineering, its potential in the field of bone defect repair has gradually emerged, becoming an important direction for solving bone repair issues in the future. By combining biological principles with engineering techniques, bone tissue engineering seeks to create functional scaffolds that promote osteogenesis, angiogenesis, and biomimetic bone regeneration. Among various materials explored for scaffold fabrication, silk fibroin, a natural polymer derived from *Bombyx mori* silkworms, has garnered significant attention due to its unique combination of biocompatibility, biodegradability, mechanical tunability, and ease of processing. These attributes make silk fibroin an excellent candidate for scaffold materials in bone tissue engineering. Silk fibroin-based scaffolds can be engineered into various forms, including membranes, hydrogels, and three-dimensional (3D)-printed constructs, to cater to diverse bone defect conditions (Figure 1). Moreover, silk fibroin can be chemically modified or combined with other biomaterials to enhance its biological and mechanical properties, making it suitable for complex bone repair scenarios. Recent studies have also demonstrated its potential in modulating cellular responses, such as osteogenic differentiation and angiogenesis, further validating its applicability in bone regeneration. This review aims to provide a comprehensive overview of recent progress in silk fibroin and its composites as scaffold materials in bone tissue engineering. The introduction begins with a discussion of the biological significance of bone and the challenges associated with current bone repair approaches. It then explores the intrinsic properties of silk fibroin that make it a promising biomaterial for bone repair applications. By critically analyzing advancements in silk fibroin-based scaffold fabrication, modification, and application, this review seeks to highlight the current status, challenges, and future prospects of silk fibroin in the field of bone repair materials.

## 2. Overview of Bone Tissue

### 2.1. Classification of Bone Tissue

Bone tissue, based on its structural composition, primarily comprises two types: cortical bone and trabecular bone, with cortical bone constituting approximately 80% of the total bone mass. It is characterized by its stiffness and relatively low porosity, typically ranging between 5% and 10%. The cortical bone is predominant in the diaphysis of long bones and provides mechanical strength and structural support. Mechanical testing of human cortical bone has revealed an average compressive strength of 105 MPa along the longitudinal axis and 131 MPa along the transverse axis. Tensile testing further determined an average longitudinal tensile strength of 53 MPa, reflecting its anisotropic mechanical properties [5]. In contrast, trabecular bone constitutes the inner portion of bones and exhibits a highly porous network structure, with porosity levels ranging from 50% to 95% [6]. This significant porosity contributes to the bone’s reduced density but provides a larger surface area compared to cortical bone, facilitating biological functions such as vascularization and hematopoiesis. The interconnected trabecular network supports the distribution of mechanical loads while housing blood vessels and bone marrow within its matrix. Structurally, intact bone integrates both cortical and trabecular components, with cortical bone forming the dense, protective external layer and trabecular bone occupying the internal regions. The compact cortical bone contains Haversian systems (also known as osteons), which are microscopic cylindrical structures facilitating vascular and nutrient supply. These are connected laterally by Volkmann’s canals, forming an intricate vascular network critical for bone metabolism and repair [7]. The distinct structural and functional characteristics of cortical and trabecular bone highlight the intricate nature of bone tissue and underscore the necessity to account for these variations when designing and applying bone repair materials.

### 2.2. Bone Tissue Composition and Structure

Bone is a highly specialized tissue with a hierarchical organization that spans multiple scales, from macroscopic structures to molecular arrangements [8]. Its unique composition and structure enable it to achieve a balance between strength and flexibility, which is critical for supporting the body and withstanding mechanical loads. Bone is primarily composed of 65 wt% inorganic components, 25 wt% organic components, 10 wt% water, and lipids [9]. The inorganic phase, predominantly hydroxyapatite, contributes to the compressive strength and stiffness of bone, while the organic matrix, mainly type I collagen, provides tensile strength and elasticity. Beyond its major components, bone also contains trace amounts of type V collagen and various non-collagenous proteins, such as osteocalcin, osteopontin, fibronectin, and bone sialoproteins, which play vital roles in bone mineralization, cell signaling, and structural integrity. The porous architecture of bone, coupled with its light weight, allows for nutrient exchange and mechanical stresses, contributing to bone’s dynamic remodeling capacity. Moreover, the composition and mechanical properties of bone are influenced by factors such as age, sex, species, and pathological conditions [10,11]. This intricate interplay of inorganic and organic components, alongside environmental factors, underscores the complexity of bone tissue and highlights the importance of developing biomimetic materials in bone tissue engineering.

### 2.3. Bone Tissue Healing Mechanisms and Repair Materials

Although bones have some inherent capacity to heal after injury, this process necessitates a series of sequential events for successful bone regeneration. Upon damage, the disruption of blood vessels leads to a significant decrease in oxygen supply around the injury site, resulting in acute necrosis of cells and severe hypoxia in the surrounding tissues [12]. In response, osteoblasts stimulate the proliferation of endothelial cells and secrete osteoblast growth factors [13,14].

Normally, endochondral ossification and intramembranous ossification are the two mechanisms of bone repair and regeneration [15]. Endochondral ossification is a relatively complex mechanism, primarily achieving bone tissue regeneration through the formation and replacement of a cartilage template [16]. First, mesenchymal stem cells (MSCs) gather at the site of injury and differentiate into chondrocytes. The chondrocytes then secrete extracellular matrix to form a cartilage template. Subsequently, chondrocytes further proliferate, become hypertrophic, and secrete vascular endothelial growth factor and type X collagen, inducing angiogenesis and the formation of mineralized tissue. Finally, after vascular invasion, chondrocytes are gradually replaced by bone tissue, ultimately forming bone tissue. Intramembranous ossification is a more direct ossification process that mainly occurs in the periosteum. Mesenchymal stem cells directly differentiate into osteoblasts [17].

Currently, bone tissue engineering materials include inert biomaterials (mainly synthetic polymers) and bioactive materials (mainly natural polymers). Bone tissue engineering materials provide a specific environment for bone tissue regeneration and facilitate the linkage between individual cells, which promotes regeneration and repair of damaged areas. Excellent bone tissue engineering materials need to have the following excellent properties: (1) excellent biocompatibility—the material will not cause inflammation, cytotoxicity, and immunogenicity after implantation; (2) suitable mechanical properties—the material can meet the support needs after implantation but will not produce the phenomenon of stress masking; (3) suitable porosity—suitable porosity is conducive to the proliferation and differentiation of the cells and the vascularization of the scaffold inside the process; (4) good osteoinductivity and conductivity; and (5) appropriate degradation rate: the material degradation rate should match the new bone growth rate.

Owing to the diverse and advantageous properties of silk fibroin, it has been extensively utilized in bone tissue engineering research. In recent years, a considerable body of research has investigated its potential applications in bone tissue regeneration. This paper provides an overview of the advancements in silk fibroin-based research within bone tissue engineering.

## 3. Characteristics of Silk Fibroin Biomaterials

The intrinsic properties of the material can substantially influence cellular behavior on biomaterials. For instance, characteristics such as molecular structure, mechanical properties, and surface morphology can affect cell adhesion, proliferation, migration, differentiation, and signaling [18,19,20]. This chapter will provide an in-depth examination of various properties of silk fibroin biomaterials.

### 3.1. Composition and Structure of Silk Fibroin

Silk fibroin is a natural high-molecular-weight fiber protein primarily derived from the silk produced by *Bombyx mori* silkworm. Its content accounts for about 70% to 80% of silk and is rich in 18 amino acids. At present, it is generally believed that silk fibroin is mainly composed of H chain, L chain, and P25 glycoprotein, with the molecular ratio of H:L:P25 = 6:6:1 [21]. The H and L chains are interconnected by disulfide bonds at their respective C termini to form a complex. In contrast, the P25 glycoprotein joins the complex in a non-covalent bond to form the basic unit of silk fibroin.

The mechanical properties of silk fibroin are primarily determined by its intrinsic molecular structure and intermolecular interactions. Among them, *β*-folding plays a key role in material strength [22]. Compared with natural silk fibers, scaffolds made from aqueous solutions of regenerated silk fibroin are relatively weak in terms of mechanical properties. The tensile strength and elongation at the break of natural silk fibroin fibers are about 0.5–0.6 GPa and 10–40% [22]. The tensile strength and elongation at the break of sericin membranes made from aqueous solutions of regenerated silk fibroin are about 0.02 GPa and 2% [23]. It was found that the number of *β*-folding of regenerated silk fibroin would increase after induction of regeneration by methanol or ethanol, improving the strength of silk fibroin-based scaffolds. For instance, the treatment of regenerated silk fibroin membranes with a methanol solution results in an increase in the *β*-sheet infrared absorption band with prolonged soaking time while concurrently decreasing the α-helix structure, thus enabling precise control over their mechanical properties [24].

### 3.2. The Characteristics of Silk Fibroin

#### 3.2.1. Biocompatibility

*Bombyx mori* silkworm silk fibroin is highly biocompatible due to its structure and chemical composition. The application of silk fibroin as a biomaterial was first evaluated in 1995, and studies demonstrated the attachment and growth of fibroblasts on the *Bombyx mori* silkworm silk fibroin material [25]. It has also been shown that cells exhibit a negligible inflammatory response to the *Bombyx mori* silkworm silk fibroin material and are highly compatible with blood [26,27,28]. This ensures that silk fibroin as a scaffold material will not cause adverse reactions in the organism itself, thereby improving the biosafety of silk fibroin scaffolds and facilitating their promotion and application as biomaterials.

#### 3.2.2. Degradability

The degradability of the biomaterial itself also plays a vital role in bone repair. The scaffold should degrade at a controlled rate according to the repair of the bone tissue damage, and its degradation products should not adversely affect the organism and should assist in forming new bone. Therefore, the degradation rate and degradation products are essential for bone repair.

When silk fibroin is degraded, the resulting amino acids can be taken up outside the body without significant toxicity to the cells [29], a property that is favorable for biomedical applications. Meanwhile, the degradation process of silk fibroin is affected by the content of both water-insoluble silk II and water-soluble silk I in their structures. When the content of silk II is increased, the number of *β*-folds of silk fibroin-based biomaterials will increase, and the degradation time will also increase [30], which proves that the degradation rate of silk fibroin-based biomaterials is controllable. The degradation rate of silk fibroin-based biomaterials can be controlled by controlling the structural content of both silk II and silk I in silk fibroin to achieve precisely regulated repair according to the damage of the target bone tissue, and the degradation products will not cause adverse reactions to the organism itself.

### 3.3. Response to Osteogenic Signaling

Although the silk protein-based scaffold does not contain any cell-specific signal expression in its primary sequence, it provides a suitable matrix for supporting the proliferation and adhesion of mesenchymal stem cells [31,32]. Moreover, the study also found that silk fibroin hydrolysates can enhance the activity of alkaline phosphatase (ALP) in osteoblasts and their ability to undergo osteogenic differentiation [33]. Significant mineral deposition was observed when silk fibroin was co-cultured with rat bone marrow mesenchymal stem cells (BMSCs) in vitro for 12 days. These results indicate that silk fibroin can trigger Wnt/*β*-catenin signaling pathway-dependent Runx2 expression in osteoprogenitor cells without dexamethasone additives [34]. According to pharmacological and molecular biology studies, Notch signaling is crucial in differentiating mesenchymal stem cells into osteoblasts [35]. Silk fibroin can promote the upregulation of osteoblast differentiation markers (such as ALP, Osterix, and Runx2) by inhibiting Notch signaling [36]. This suggests that silk fibroin-based scaffolds can enhance the transmission of osteogenic-related signals, thereby aiding in the repair of bone defects.

In summary, owing to its unique mechanical properties, biocompatibility, biodegradability, and osteogenic signaling capabilities, silk fibroin exhibits significant potential as a bone repair scaffold material. This provides a robust theoretical foundation for the broad application of silk fibroin-based biomedical materials.

## 4. Current Status of Silk Protein Applications in Bone Repair

Silk fibroin is a natural biomaterial with excellent biocompatibility and mechanical properties. It can be engineered into various materials through multiple processing techniques, including sponge-like porous scaffolds, hydrogels, electrospun fibers, and 3D-printed scaffolds. These materials have wide applications in bone tissue engineering, and the most suitable silk fibroin-based material can be selected based on the specific characteristics of the bone defect to promote rapid healing of the defect site. For details, please refer to Table 1.

### 4.1. Silk Fibroin Scaffold Bone Repair Materials

#### 4.1.1. Silk Fibroin Sponge-like Porous Scaffold Bone Repair

Silk fibroin sponge-like porous scaffolds are prepared through methods such as freeze-drying, salt leaching, and chemical crosslinking [38,62,63]. They possess advantages such as high porosity and good mechanical properties, which can promote cell attachment and growth. The porous structure of the scaffold is similar to a sponge, with excellent water absorption properties, allowing it to quickly absorb large amounts of liquid. Compared to hydrogels, silk fibroin sponge-like porous scaffolds exhibit better mechanical properties, such as higher compressive strength and elastic modulus, making them more stable and reliable under physiological loads. In preclinical studies of bone repair, silk fibroin is frequently combined with other materials that promote osteogenesis for bone regeneration. Hydroxyapatite, a natural apatite mineral, has the capacity to interact with bone marrow mesenchymal stem cells (BMSCs) and enhance BMSC proliferation, differentiation, and mineralization through its action on various signaling pathways, including ERK/Sox9, BMP/Smad, Wnt/*β*-catenin, TGF-β, and MAPK [64,65,66,67,68]. Studies have found that hydroxyapatite, after being alternately soaked or directly deposited in CaCl_2_ and Na_2_HPO_4_ solutions, is remixed with regenerated silk fibroin solutions to form a porous silk fibroin scaffold [69,70]. Compared with the uncombined silk fibroin scaffold, the composite porous fibroin scaffold can form more bone tissue faster. Some scholars have prepared a composite bionic scaffold of silk fibroin/chitosan/nano-hydroxyapatite combined with autologous concentrated growth factor (CGF) and found that this composite scaffold is more conducive to the adhesion, proliferation, and osteogenic differentiation of bone marrow mesenchymal stem cells (BMSCs). The results of animal experiments show that the composite scaffold has higher efficiency in repairing bone defects [71]. In addition to HA, other biomaterials form composites with silk fibroin that exhibit osteoconductivity, such as chitosan and aloe vera. The composite of silk fibroin and other biomaterials that are beneficial to bone regeneration can produce composite scaffolds that are more conducive to bone damage repair, and this synergistic effect of ‘1 + 1 > 2’ provides a new idea for the research and development of scaffolds for bone defect repair and effectively improves the efficiency of bone repair.

#### 4.1.2. 3D-Printed Silk Fibroin Scaffold

Existing experimental design and manufacturing techniques are inadequate in precisely controlling the material’s re-absorption properties and frequently lack a predefined internal architecture. This results in the random pore structures of most produced scaffolds, which lack a well-defined internal framework. Such randomness interferes with normal blood vessel growth, leading to suboptimal osseointegration within the scaffold. In contrast, 3D bioprinting technology enables the precise free-form fabrication of scaffolds with predefined internal structures, effectively addressing these limitations.

3D bioprinting utilizes computer-aided design to precisely position target cells, which are subsequently printed individually or in layers to construct scaffolds. A research team employed low-temperature extrusion 3D-printing technology to fabricate a porous hydrogel scaffold through horseradish peroxidase (HRP)-mediated cross-linking of silk fibroin and tyramine-modified gelatin (GT). This process, combined with stem cell polymerization and somatic inoculation, promoted the elevated expression of type II collagen, thereby enhancing the regenerative repair of articular cartilage. This suggests that 3D-printed SF-GT hydrogel has a promising clinical application in promoting cartilage regenerative repair [72]. Furthermore, the investigators synthesized nanohydroxyapatite (nHA)/methacrylic acid cellulose (MASF) composite scaffolds utilizing light-curing technology in conjunction with three-dimensional printing. The constructed nHA/MASF biocomposite exhibits good biocompatibility and excellent osteogenic function [73].

The aforementioned findings demonstrate that the silk fibroin scaffold material fabricated via 3D-printing technology exhibits excellent performance in bone repair applications. It can be precisely molded to match the specific geometry of the bone defect site, thereby achieving accurate and effective repair of the defect.

#### 4.1.3. Electrospun Silk Fibroin Scaffold

In recent years, as electrospinning technology has progressively matured, the fabrication of silk fibroin nanofibers via electrospinning has emerged as a focal point in the development of silk fibroin materials. The diameter of nanofibers produced through this method closely resembles that of fibronectin in the extracellular matrix (for instance, some researchers have achieved fibers with an average diameter of 80 nm using formic acid as a solvent [74]), thereby more accurately mimicking the structure of the extracellular matrix. This similarity significantly enhances the efficiency of stem cell growth, differentiation, and extracellular signaling on the material. Consequently, electrospinning represents an advanced approach to developing and utilizing silk fibroin.

The researchers used the three-dimensional porous scaffolds of silk fibroin prepared by electrostatic spinning and three-dimensional PLA scaffolds to carry out experimental comparative studies, and the results showed that the silk fibroin scaffolds could significantly promote the ALP activity and osteogenic differentiation ability of mouse embryonic osteoblast precursor cells (MC3T3-E1); moreover, the silk fibroin scaffolds were significantly superior to the PLA scaffolds in terms of the ability to form new bone in the animal experiments [75], which suggests that electrostatically spun silk fibroin scaffolds have good potential for bone tissue repair. Researchers have also prepared a silk-based scaffold with a three-layer structure (silk fibroin doped with zinc oxide nanoparticles, pure silk fibroin, and silk fibroin doped with hydroxyapatite nanoparticles) for bone repair using a continuous electrospinning method, demonstrating good biocompatibility and enhanced osteogenic and antibacterial properties [76].

#### 4.1.4. Silk Fibroin Hydrogel Bone Repair Material

Silk fibroin hydrogels can be formed by changing the pH of the solution, by using dehydrating agents, or by transforming the aqueous solution of silk fibroin into a gel under different conditions, such as ultrasound. If the silk fibroin solution’s temperature, concentration, Ca^2+^ concentration, or ultrasound frequency is changed under the same conditions, it can change the silk fibroin gelation process [77,78]. Despite the poor mechanical properties of silk protein-based hydrogels, its inherent fluidity and injectability make it an ideal choice for repairing irregularly shaped bone defects. At the same time, it can be used as a delivery medium and combined with other bone repair materials to achieve better bone defect repair results. Researchers have prepared injectable hydrogels using ultrasound methods for the sustained release of vascular endothelial growth factor and bone morphogenetic protein-2; animal experiments showed that by injecting into the rabbit’s maxillary sinus floor between the mucous membrane and the bone wall for 12 weeks, there was a new bone formation in the maxillary sinus floor of the rabbits in the experimental group. This result benefits those developing surgeries such as oral implants [79]. In addition, research teams have constructed a bilayer silk fibroin scaffold for osteochondral repair. A dense and smooth bionic cartilage layer (D/S) was used to mimic the surface properties of natural cartilage, a porous layer (P/S) loaded with BMP-2 was used to promote the regeneration of bone tissue, and the edges of the scaffold were filled and sealed with a light-curing hydrogel (Sil-MA) loaded with TGF-β3. This design solves the bone–cartilage connection problem and achieves horizontal integration of the newborn cartilage with the surrounding natural cartilage through a light-cured hydrogel. This integrated double-layer scaffold combined with the edge-sealing strategy of the light-curing hydrogel can effectively promote the regeneration of osteochondral cartilage and achieve good integration of the newborn cartilage with the surrounding natural cartilage, which is a potential strategy for osteochondral repair [80].

#### 4.1.5. Silk Fibroin Membrane Bone Repair Material

Silk fibroin membranes are formed by dissolving silk fibroin in water or other organic solvents, followed by cooling and drying them in a static mold. By exposing the silk fibroin membranes to water or methanol vapor, the stability of the silk fibroin film can also be improved, which will increase the β-sheet content of the silk fibroin, thereby enhancing the stability of the silk fibroin film [81].

In one study, silk was dissolved using a CaCl_2_ solution and ethanol-induced silk fibroin to form more *β*-folds, which was prepared into a more stable silk fibroin membrane. It was used to repair rabbit cranial defects, and it was found that using silk fibroin membrane could promote the regeneration of new bone. Although silk protein membranes have better biocompatibility, degradability, and toughness, their clinical application in significant bone defects is severely limited due to their poor mechanical properties. Composite bone induction membranes are usually prepared by combining with other active substances to meet the requirements of mechanical properties. For example, the silk fibroin composite membrane was prepared using biologically active calcium silicate particles. This composite membrane not only improved the brittle characteristics of traditional silk fibroin membranes but also promoted the proliferation and adhesion of MG63 cells due to its good hydrophilicity and biocompatibility, providing beneficial implications for the application of silk fibroin membrane in bone tissue engineering [82]. Researchers used a green papermaking method to prepare a silk fibroin/collagen hybrid membrane. This membrane combines the fibrous, sponge, and film forms of silk fibroin to design a single-material bone regeneration membrane that is multi-structured yet well-integrated. This multi-form SF membrane shows great potential in guided bone regeneration applications [83].

## 5. Summary and Outlook

Silk fibroin is a natural, high-molecular-weight fibrous protein derived from silk. Its remarkable biocompatibility, biodegradability, adjustable mechanical properties, and ease of processing have attracted considerable interest in bone tissue engineering. It can be fabricated into several scaffold configurations, including membranes; hydrogels; and 3D-printed, electrospun, and sponge-like porous scaffolds, to address various types of bone deformities. Moreover, silk fibroin can improve its efficacy when integrated with other biomaterials, rendering it appropriate for intricate bone repair situations. It demonstrates superior capability compared to pure silk fibroin scaffolds in modulating osteogenic differentiation and angiogenesis. This paper offers an extensive examination of silk fibroin and its composite materials in bone tissue engineering, emphasizing preclinical investigations of diverse silk fibroin-based composite scaffolds in osteogenesis. Due to the complexity of human bone repair, the clinical translation of silk fibroin scaffolds for bone repair faces some challenges. These include developing silk fibroin scaffolds with enhanced mechanical strength to withstand physiological loads, precisely controlling the degradation rate to synchronize with new bone formation, and improving the compatibility and integration of silk fibroin with other biomaterials to minimize potential toxic side effects. In future research, efforts should focus on deeply exploring the properties of silk fibroin, enhancing manufacturing processes, and improving functional modification strategies to ensure its performance can precisely meet specific clinical needs. As progress continues in these areas, it is anticipated that silk fibroin-based biomaterials will evolve into versatile, high-performance solutions capable of meeting the complex requirements of bone tissue repair and regeneration in clinical settings.

## Figures and Tables

**Table 1:** Research on different types of silk fibroin-based composite scaffolds and their applications in animal models.

Types of Silk Fibroin-Based Composite Scaffolds	Main Components of Silk Fibroin Composite Scaffolds	Animal Models	Ref
sponge-like porous scaffold	Silk fibroin, CaP	Rat femur	[37]
sponge-like porous scaffold	Silk fibroin, collagen	Rabbit cartilage	[38]
sponge-like porous scaffold	Silk fibroin, β-tricalcium phosphate, Bone morphogenetic protein-2	Rabbit left radius	[39]
sponge-like porous scaffold	Silk fibroin, Bone morphogenetic protein-2	Mouse skull	[40]
sponge-like porous scaffold	placental-derived extracellular matrix	Rabbit tibia	[41]
3D printing	Silk fibroin, Silk fibroin, Collagen, Hydroxyapatite, Recombinant human erythropoietin	Rabbit alveolar bone	[42]
3D printing	Silk fibroin, Gelatin	Rabbit articular cartilage	[43]
3D printing	Silk fibroin, Cellulose, Chitosan	Rat skull	[44]
3D printing	Silk fibroin, Polycaprolactone	Rabbit skull	[45]
3D printing	Silk fibroin, Polylactic acid, Hydroxyapatite	Rat femur	[46]
Electrospun	Silk fibroin, Lactide-co-ε-caprolactone, Human adipose-derived stem cells	Rat femur	[47]
Electrospun	Silk fibroin, Hydroxyapatite, BMP-2	Rat skull	[48]
Electrospun	Silk fibroin, Bioactive glass, Collagen	Rat tibia	[49]
Electrospun	Silk fibroin, Hydroxyapatite, Polydopamine	Mouse skull	[50]
Electrospun	Silk fibroin, Graphene oxide, BMP-2	Rat skull	[51]
Hydrogel	Silk fibroin, NapFFRGD	Mouse skull	[52]
Hydrogel	Silk fibroin, Bioactive glass, Chitosan	Rat skull	[53]
Hydrogel	Silk fibroin, Tannic Acid, Fe_3_O_4_ nanoparticles	Rat skull	[54]
Hydrogel	Silk fibroin, LPONITE	Rat skull	[55]
Hydrogel	Silk fibroin, MXene	Rat skull	[56]
Membrane	Silk fibroin	Rabbit skull	[57]
Membrane	Silk fibroin, 4-hexylresorcinol	Rabbit skull	[58]
Membrane	Silk fibroin, Tetracycline	Rat skull	[59]
Membrane	Silk Fibroin, Collagen	Subcutaneous in rats	[60]
Membrane	Silk Fibroin, Tetracycline	Rabbit skull	[61]
